# The effect of gamification on the medication knowledge, performance and satisfaction of nurses in continued medical education: A quasi-experimental study

**DOI:** 10.1371/journal.pone.0331372

**Published:** 2025-09-04

**Authors:** Raziyeh Ghafouri, Shiva Ghasemniaye Namaghi, Banafshe Khoshgoui

**Affiliations:** 1 Department of Medical Surgical Nursing, School of Nursing & Midwifery, Taleghani Hospital Research Development Committee, Shahid Beheshti University of Medical Sciences, Tehran, Iran; 2 Ayatollah Taleghani Hospital, Shahid Beheshti University of Medical Sciences, Tehran, Iran; 3 Taleghani Hospital Research Development Committee, Shahid Beheshti University of Medical Sciences, Tehran, Iran; Farhangian Teacher Education University: Farhangian University, IRAN, ISLAMIC REPUBLIC OF

## Abstract

**Background and objective:**

One of the primary challenges within the health system is improving nurses’ motivation and attitude toward participation in the continuous education program. This study was conducted aim to investigate the effect of gamification on medication knowledge, performance and satisfaction of nurses in continued medical education (CME).

**Methodology:**

The study was conducted as a quasi-experimental design from November 1, 2022, to February 1, 2024. Participants were 128 nurses with a minimum of 6 months of work experience who were randomly assigned to two groups, intervention and control groups via a colored card. Baseline assessments of medication knowledge and drug administration practices were conducted one week prior to the intervention. Education in both groups consisted of five 2-hour sessions. In the control group, education was delivered using the lecture method, while in the intervention group, the competitive software (Kahoot!) was used. One week after the completion of the training sessions, participants’ knowledge, drug administration skills, and satisfaction were evaluated.

**Results:**

The participants’ average (standard deviation) age was 30.34 (5.34), with 49 male (38.35%) and 79 female (61.7%). Wilcoxon signed-rank test results demonstrated statistically significant difference in the knowledge and performance within both groups (p < 0.001). Mann-U-Whitney test findings revealed a significant difference between the two groups of participants in performance and satisfaction with the educational approach education (p < 0.001).

**Conclusion:**

The gamification method enhanced nurses’ satisfaction and medication performance. Using Kahoot as a game-based competitive application has been shown to enhance nurses’ knowledge of pharmacology as well as increase participants’ satisfaction with educational programs. Therefore, it is recommended that nursing educators and administrators integrate gamified approaches and strategies—such as Kahoot!—to enrich learning experiences and motivate participation in ongoing professional development.

## Introduction

Medication administration constitutes a fundamental responsibility within nursing practice [1]. To ensure safe and effective pharmacological treatment, nurses must administer the correct medication to the correct patient, in the correct dosage, with the correct route, and mitigate adverse effects [2,3]. In other words, it requires cognitive, practical, interpersonal, ethical, and legal performances in order to have the ability to calculate, prepare, and monitor the side effects and interactions of drugs [4,5]. Therefore, nurses need theoretical knowledge and the ability to make decisions and judgments regarding drug interactions, possible side effects, and side effect monitoring and prevention [1,6,7].

Numerous factors play a role in nurses’ medication performances, which include the nurse’s knowledge of calculation, drug interactions and side effects, drug administration and monitoring performances, as well as the nurse’s professional attitude and commitment to report medication errors [8,9]. Given that the main factors are in the fields of knowledge, attitude, and performance of nurses, it is necessary to reform educational programs to improve the knowledge, attitude, and behavioral performances of nurses. Such measures may lead to decreased medication errors and improved patient safety [6,10]. To achieve this goal, it is necessary to consider appropriate programs, including continuous education, to improve the knowledge, attitude, and performance of giving medicine by nurses in order to prevent errors in this process [11–13].

Game-based education is an educational method that uses game elements to teach learners a specific skill [13,14]. In gamification, like game-based education, it is the use of game elements and their combination with educational content [15,16]. Gamification, like game-based education, education and games are completely interwoven, but gamification has an additional layer on the content [15,17,18]. In gamification, by giving rewards and prizes such as badges, it encourages the user (learner) to use more and strengthens the learning goals [16,17]. In gamification, learning goals will be strengthened in learners with a sense of competition in evaluation and receiving rewards and points [17,18].

Kahoot as a game-based platform aligned with intrinsic motivation theory. Based on the intrinsic motivation theory, three categories of factors make learning fun: 1) challenge (goals with uncertain outcomes), 2) fantasy (pervasive engagement through internal or external fantasy), and 3) curiosity (creating a sense of curiosity through graphics and sound and cognitive curiosity) [16]. So, the present research was conducted to investigate the effect of gamification on the medication knowledge, performance, and satisfaction of nurses in continued medical education.

## Methods

The study was conducted as a quasi-experimental design from November 1, 2022, to February 1, 2024.

### Participants

The participants were selected by the convenience method. All the nurses who worked in the internal medicine, surgery, gastroenterology, orthopedics, and hematology departments were 180 nurses. The inclusion criteria were to have a nursing degree with six months of clinical experience and willingness to participate in the research. The exclusion criteria included non-attendance in all educational sessions and not completing the questionnaire completely and any nurses on holiday or absent due to illness. The number of required samples per group was calculated using the following formula:


$$n\geq 2\frac{{{(z}_{\alpha /2}+z_{\beta })}^{2}\sigma^{2}}{({\mu_{1}-\mu_{2})}^{2}}$$

The effect size was derived from the study by Khaledi and colleagues [13]. Taking into account a 60% potential dropout rate, a minimum of 70 participants was considered for each group.


$$n=2(1.96+0.85{)}^{2}{\left(\frac{1}{0.50}\right)}^{2}\;(1-0.3)=44$$

**Fig 1:**
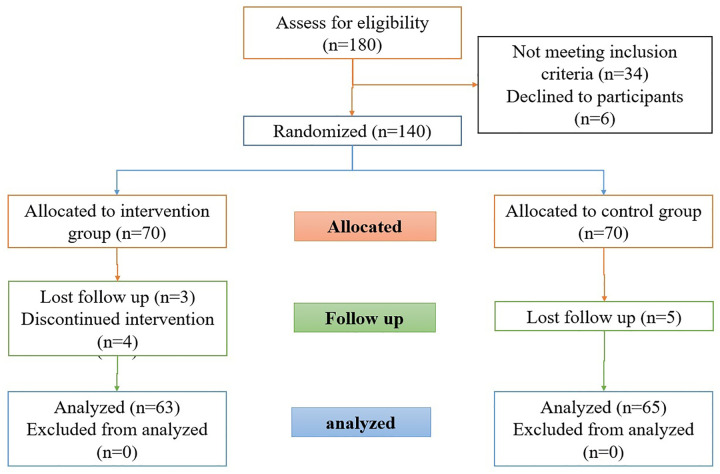
CONSORT flow diagram od research.

The exclusion criteria included completing the questionnaire incompletely. Following the application of the predefined inclusion criteria, 34 individuals were excluded from the outset due to having less than six months of clinical work experience. Furthermore, an additional 6 individual declined to participate in the study, resulting in their exclusion from the final samples. In total, 128 nurses participated in this study. Group allocation was performed using colored cards. Prior to the start of the study, 140 colored cards (70 red and 70 blue) were prepared. Before intervention, participants randomly selected one of the cards. The sampling process is shown in Fig 1.

### Data collection

After completing the informed consent form, an educational needs assessment was done for all the participants, and that was the same for both groups. In this stage, medication calculations, knowledge, attitude, and performance of nurses were investigated, and the educational needs of participants were identified. One week prior to the start of the study, participants’ knowledge and drug administration practices were assessed using the research tools. Then, according to educational needs, educational goals were formulated, and based on the goals, educational content (relying on pharmaceutical knowledge, drug interactions, drug calculation performances, drug prescription, patient monitoring, and patient drug education) was prepared.

Education in both groups consisted of five 2-hour sessions. In the control group, education was delivered using the lecture method, while in the intervention group, the competitive software (Kahoot!) was used. In the intervention group, after the lecture-style training, 15 minutes were allocated to playing Kahoot. Participants accessed the game using their mobile phones and selected their answers on their devices after each question was presented. Participants play the game individually. For each section, 3 competitions were designed on the Kahoot platform. The first competition was about medication preparation, the second was about medication for resuscitation nursing intervention, and the last was about drug interactions. All the competition was based on scenario-based questions. One week after the completion of the training sessions, participants’ knowledge, drug administration practices, and satisfaction were evaluated using the study instruments.

Participants’ medication knowledge and performance of two groups were evaluated before and after the intervention. In addition, satisfaction was evaluated only post-intervention in both groups and compared between the two groups. Due to the use of Kahoot for training in the intervention group, it was not possible to blind the participants. Therefore, the statistical analysis and the collector of the outcome questionnaires were blinded and did not know about the grouping.

### Data collection tools

The study tools included a demographic information questionnaire, the best evidence tool for medication that was used by Salami and colleagues in their research [19], and an education satisfaction questionnaire. The demographic information questionnaire included age, gender, education level, and service department. The best evidence tool for medication and the education satisfaction questionnaire each contained 10 questions designed based on Likert’s five-point score. For both of the questionnaires, the final score was calculated.

The best evidence tool was first translated and back translated, and then its validity was assessed [20,21]. To assess the instrument’s validity, an expert panel was formed consisting of six nursing faculty members with at least six years of teaching experience and four clinical nurses with over fifteen years of clinical management experience. In the initial phase, the panel evaluated the face validity of the questionnaire. During this stage, the wording of the items was revised, although no items were removed.

Subsequently, content validity was assessed using the Content Validity Index (CVI) and Content Validity Ratio (CVR). The content validity index was higher than 0.7 regarding the simplicity, ambiguity, and relevance of each item in both questionnaires. The CVR was calculated based on the responses to the necessity of questions (nE) (CVR=(nE-N/2)/ (N/2)). The minimum acceptable CVR based on the number of professors participating in the validity review (at least 10) was 0.49, according to Lawshe’s table [20,21].

In this study, CVR was higher than 6.0 in both questionnaires. Cronbach’s alpha was used for the instrument’s reliability [20,21]. Cronbach’s alpha lower than 0.3 was considered low reliability, between 0.3 and 0.7 as fair, and more than 0.7 as good reliability [21]. In this study, Cronbach’s alpha of the best evidence tool was 0.82, and Cronbach’s alpha of education satisfaction was 0.80.

## Data analysis

Data analysis was performed using the Statistical Package for the Social Sciences (SPSS), version 20, (IBM Corp, Amonk, New York). Descriptive statistics, including mean, standard deviation, and percentage, were used. To compare the results before and after the intervention, the Wilcoxon signed-rank test was used, and to compare the results between the two groups, the Mann–Whitney U test and Chi- square was used. A significant level in all statistical tests was considered 0.05.

### Ethics, approval, and consent to participate

This study was approved by the Ethics Committee of Shahid Beheshti University of Medical Sciences. Accordingly, written informed consent was obtained from all participants after providing a clear explanation of the study’s objectives. Participants were assured of their right to freely participate in or withdraw from the research at any stage. All research methods were carried out in accordance with the research ethical codes of the Iranian National Committee for Biomedical Research (ethical code: IR.SBMU.RETECH.REC.1401.239) at July 24, 2022 and it is available on: https://ethics.research.ac.ir/ProposalCertificateEn.php?id=271066&Print=true&NoPrintHeader=true&NoPrintFooter=true&NoPrintPageBorder=true&LetterPrint=true.

## Results

In total, 128 nurses from the internal medicine, surgery, gastroenterology, orthopedics, and hematology departments participated. In addition, 49 people (38.3%) were men and 79 (61.7%) were women. The mean (standard deviation) age of the participants in the intervention and control groups was 30.52 (5.44) and 30.17 (5.29) years, respectively. The demographic information of the participants is presented in Table 1.

**Table 1:** The demographic finding of the participants.

Demographic Characters		Group				Result
		Intervention		Control		
		Count	N %	Count	N %	
**Department**	**Hematology**	8	6.3%	8	6.3%	χ^2^ = 0.04 df = 3 p = 0.99
	**Surgery**	10	7.8%	11	8.6%	
	**Orthopedic**	17	13.3%	18	14.1%	
	**Gastroenterology**	28	21.9%	28	21.9%	
**Gender**	**Male**	24	18.8%	25	19.5%	χ^2^ = 0.002 df = 1 p = 0.96
	**Female**	39	30.5%	40	31.3%	
**Marriage**	**Marriage**	25	19.5%	21	16.4%	χ^2^ = 1.92 df = 2 p = 0.38
	**Single**	37	28.9%	44	34.4%	
	**Widow**	1	0.8%	0	0.0%	
**Management History**	**Supervisor**	2	1.6%	2	1.6%	χ^2^ = 0.003 df = 2 p = 0.99
	**Head Nurse**	4	3.1%	4	3.1%	
	**Nurse**	57	44.5%	59	46.1%	

The Wilcoxon signed-rank test showed that there is a significant difference between the mean of medication knowledge and performance before and after intervention (P < 0.001). Also, the Wilcoxon signed-rank test showed that there is a significant difference between the mean of medication knowledge of two groups before and after intervention, but there is a significant difference between the mean of medication performance before and after intervention in the intervention group (P < 0.001), and it was not significant in the control group (P > 0.05) (Table 2).

**Table 2:** Comparison before and after intervention (Wilcoxon signed-rank test results).

	Intervention		Control		Total	
	Mean	SD	Mean	SD	Mean	SD
**Knowledge (Before)**	18.27	3.09	18.06	2.42	18.16	2.76
**Knowledge (After)**	24.06	2.25	23.42	2.78	23.45	2.46
**Test result**	p < 0.001		p < 0.001		p < 0.001	
**Medication Performance (Before)**	19.97	4.81	20.91	4.11	20.45	4.48
**Medication Performance (After)**	25.25	3.30	21.85	3.58	23.52	3.84
**Test result**	p < 0.001		p = 0.09		p < 0.001	

The Mann-U-Whitney test showed that there is a significant difference between the two groups of participants in terms of medication performance and education satisfaction after intervention (p < 0.001), but the Mann-U-Whitney test showed that there is not a significant difference between the two groups of participants after intervention in terms of knowledge (p > 0.05) (Table 3).

**Table 3:** Results of comparison between the two groups.

	Intervention			Control			Result (Mann-U-Whitney test)
	Mean	SD	Mean Rank	Mean	SD	Mean Rank	
**Knowledge (Before)**	18.27	3.09	66.95	18.06	2.42	62.12	p = 0.46
**Knowledge (After)**	24.06	2.25	68.85	23.42	2.78	60.28	p = 0.17
**Medication Performance (Before)**	19.97	4.81	62.29	20.91	4.11	66.64	p = 0.50
**Medication Performance (After)**	25.25	3.30	81.48	21.85	3.58	48.04	p < 0.001
**Satisfaction (After)**	24.75	4.06	80.01	20.94	4.55	49.47	p < 0.001

## Discussion

This study was conducted to evaluate the effects of gamification on nurses’ medication knowledge, performance, and satisfaction of nurses in continued medical education. The results of this study indicated a statistically significant difference between the intervention and control groups of participants in terms of nurses’ medication performance, and satisfaction with education (p < 0.001).

Previous research supported these findings. For instance, Gockian and colleagues investigated the effects of gamification on the dermatology knowledge of medical science undergraduate students and found that it enhanced learners**’** enthusiasm for the education process [22].

Similarly, Ismail and colleagues conducted a cognitive phenomenological study utilizing Kahoot software, which demonstrated that game-based learning significantly increased students’ motivation [23]. Backhouse and colleagues applied game elements to simulate patient safety for medical students, noting elevated educational satisfaction and self-confidence in safety-related competency [24]. These findings align with the present study and reinforce the notion that gamification is an effective pedagogical tool for adult learners and professionals.

Additionally, a paired t-test demonstrated a significant improvement in the performance before and after the education (P < 0.05). Ali and Abdalgane reported that Kahoot enhanced vocabulary acquisition in English language learners by fostering motivation, positive emotional engagement, and satisfaction with the learning environment [15].

Moreover, games are a socially centered process and can enhance motivation and learning across all levels and ages [16,18]. Platforms such as Kahoot prove particularly valuable for continuous education. The findings of the Tan Ai Lin and colleagues study showed the benefit of Kahoot in terms of: 1) inducing motivation as well as engagement, and 2) fostering and reinforcing learning (for both theoretical and practical aspects) [16]. Likewise, Wirani and colleagues highlighted Kahoot’s effectiveness in delivering gamified education through elements of competitiveness, challenge, and enjoyment [25].

Göksün & Gürsoy endorsed Kahoot’s role in promoting motivation and a sense of competition in educational settings while identifying infrastructure and internet access as potential implementation barriers [26]. Salehi and colleagues, through a systematic review, argued that the efficacy of gamification in medical education depends on cognitive, psychomotor, and affective factors [27]. Their conclusion supports the current study, which found a statistically significant advantage for the gamified instruction group.

Despite occasional resistance from participants and internet limitations, such challenges can be mitigated through targeted consultation and clear communication of benefits. This study contributes substantively to the expanding literature on gamification in nursing education by implementing a rigorously structured intervention with quantifiable learning outcomes. Rooted in authentic clinical educational contexts, the research addresses practical challenges—particularly medication administration—which enhances its relevance to ongoing professional development initiatives.

The incorporation of Kahoot as a mobile-enabled gamification platform facilitates flexible instructional delivery, accommodating diverse learner preferences and extending engagement beyond conventional classroom boundaries. By proactively mitigating potential barriers, such as participant hesitation and limited internet infrastructure, the study evidences a responsive and inclusive pedagogical approach. These design features enhance the study’s reliability and promote equitable access, strengthening its applicability across varied healthcare learning environments. Importantly, the extension of gamified strategies to continuing medical education (CME) marks a critical advancement, expanding the intervention’s utility beyond pre-licensure training to encompass lifelong learning among healthcare professionals.

Empirical findings from the study indicate that gamification—particularly via platforms such as Kahoot—significantly improves both learner satisfaction and clinical competencies, including medication administration. By integrating game-informed learning strategies, educators can foster enriched environments that cultivate learner autonomy, critical thinking, and sustained engagement. Furthermore, strategies addressing learner resistance through orientation and promoting technical accessibility are essential for optimizing the efficacy of such interventions across diverse educational settings.

While this investigation illuminates the pedagogical potential of gamification, further empirical inquiry is warranted to evaluate its sustained influence on clinical performance and long-term knowledge retention. Future studies should explore behavioral outcomes that transcend cognitive gains, including collaborative teamwork, decision-making under time constraints, and adaptability in complex clinical scenarios. Comparative research analyzing alternative gamified modalities—such as simulation-based versus quiz-oriented mechanisms—may elucidate optimal instructional designs aligned with specific learning objectives. Also, develop further studies that deepen the motivations of trainees and trainers in the use of new pedagogical strategies such as gamification.

## Conclusion

The integration of gamification into nursing education has demonstrated considerable efficacy in enhancing nurses’ satisfaction and improving medication administration performance. As an innovative pedagogical strategy, gamification supports continuous medical education (CME) by fostering active engagement and motivation through interactive platforms. Specifically, tools such as Kahoot exemplify the potential of game-based systems to stimulate curiosity, promote enjoyment, and encourage a creative learning environment. In light of these findings, it is recommended that gamification be adapted not only in foundation nursing curricula but also in ongoing professional development initiatives to optimize educational outcomes and learner engagement.

### Research limitations

Every type of scientific research is associated with limitations. Like all empirical investigations, the present study was subject to inherent limitations. One of them was the initial resistance of some participants toward the gamified learning approach (same as don’t like to participate in the game), stemming from unfamiliarity or lack of interest in game-based education methods. This hesitancy was mitigated through direct communication, an orientation session, and reassurance regarding the educational value of the intervention.

Additionally, limited internet access posed a technical challenge for certain participants, potentially affecting engagement with the mobile learning platform. This was addressed by providing the necessary internet connectivity to ensure that participants have equitable access to the gamified modules. While these solutions reduced the impact of such limitations, further research should consider scalable approaches to support broader implementation across diverse educational settings.

## Supporting information

S1 Data Minimal research data. (DOC)
