# Supplementary material for: The effect of gamification on the medication knowledge, performance and satisfaction of nurses in continued medical education: A quasi-experimental study
Source: PLoS One. 2025 Sep 4;20(9):e0331372. doi: 10.1371/journal.pone.0331372 (PMC12410705; doi:10.1371/journal.pone.0331372)
Supplement: S1 Data — (DOC) [file pone.0331372.s001.doc]

Descriptive Data


	Mean	Standard Deviation	
Age Nurse	30.34	5.34	
Knowledge Before	18.16	2.76	
Knowledge After	23.73	2.55	
Medication Before	20.45	4.48	
Medication After	23.52	3.84	
Satisfaction	22.81	4.70	


	Group	
	Intervention A	Control: B	
	Mean	Standard Deviation	Mean	Standard Deviation	
Age Nurse	30.52	5.44	30.17	5.29	
Knowledge Before	18.27	3.09	18.06	2.42	
Knowledge After	24.06	2.25	23.42	2.78	
Medication Before	19.97	4.81	20.91	4.11	
Medication After	25.25	3.30	21.85	3.58	
Satisfaction After	24.75	4.06	20.94	4.55	


	Count	 N %	
Group	Intervention A	63	49.2%	
	Control: B	65	50.8%	
Ward	Hematology	16	12.5%	
	Surgery	21	16.4%	
	Arthopedic	35	27.3%	
	Digestive	56	43.8%	
Gender Nurse	Male	49	38.3%	
	Female	79	61.7%	
Marriage Nurse	Married	46	35.9%	
	Single	81	63.3%	
	Vidvo	1	0.8%	
	Divorced	0	0.0%	
Education Nurse	Diploma	8	6.3%	
	Bacleared	120	93.8%	
Management	Nurse	116	90.6%	
	Head Nurse	8	6.3%	
	Supervisor	4	3.1%	


	Group	
	intervention A	Control: B	
	Count	N %	Count	N %	
Ward	Hematology	8	6.3%	8	6.3%	
	Surgery	10	7.8%	11	8.6%	
	Orthopedic	17	13.3%	18	14.1%	
	Digestive	28	21.9%	28	21.9%	
Gender Nurse	Male	24	18.8%	25	19.5%	
	Female	39	30.5%	40	31.3%	
Marriage Nurse	Married	25	19.5%	21	16.4%	
	Single	37	28.9%	44	34.4%	
	Vidvo	1	0.8%	0	0.0%	
	Divorced	0	0.0%	0	0.0%	
Education Nurse	Diploma	8	6.3%	0	0.0%	
	Bacleared	55	43.0%	65	50.8%	
Management	Nurse	57	44.5%	59	46.1%	
	Head Nurse	4	3.1%	4	3.1%	
	Supervisor	2	1.6%	2	1.6%	


Medication performance before intervention	Count	N %	
Does the nurse perform a complete patient identification and check the patient's wristband before administering the medication? (Patient Identification)?	1.00	17	13.3%	
	2.00	41	32.0%	
	3.00	70	54.7%	
Does the nurse prepare medications for more than one patient at a time?	1.00	20	15.6%	
	2.00	68	53.1%	
	3.00	37	28.9%	
	4.00	3	2.3%	
Does the nurse label the patient's name and room number on the medication container?	1.00	26	20.3%	
	2.00	80	62.5%	
	3.00	22	17.2%	
Does the nurse complete the safe medication administration checklist for preparing the medication?	1.00	27	21.1%	
	2.00	62	48.4%	
	3.00	39	30.5%	
Does the nurse label the medication name, patient's name, and room number on the syringe and pump?	1.00	25	19.5%	
	2.00	55	43.0%	
	3.00	48	37.5%	
Does the nurse prepare the medication by two people?	1.00	22	17.2%	
	2.00	69	53.9%	
	3.00	33	25.8%	
	4.00	4	3.1%	
Does the nurse double-check the unusual amount of medication?	1.00	35	27.3%	
	2.00	67	52.3%	
	3.00	26	20.3%	
Does the nurse double-check the insulin dose?	1.00	34	26.6%	
	2.00	61	47.7%	
	3.00	31	24.2%	
	4.00	2	1.6%	
Does the nurse check the patient's allergy history before administering the medication?	1.00	35	27.3%	
	2.00	69	53.9%	
	3.00	24	18.8%	
Is another nurse present during the preparation and administration of the medication?	1.00	61	47.7%	
	2.00	57	44.5%	
	3.00	9	7.0%	
	4.00	1	0.8%	


Medication performance before intervention
	Group	
	Intervention A	Control: B	
	Count	N %	Count	N %	
Does the nurse perform a complete patient identification and check the patient's wristband before administering the medication? (Patient Identification)?	1.00	11	8.6%	6	4.7%	
	2.00	18	14.1%	23	18.0%	
	3.00	34	26.6%	36	28.1%	
Does the nurse prepare medications for more than one patient at a time?	1.00	13	10.2%	7	5.5%	
	2.00	31	24.2%	37	28.9%	
	3.00	19	14.8%	18	14.1%	
	4.00	0	0.0%	3	2.3%	
Does the nurse label the patient's name and room number on the medication container?	1.00	15	11.7%	11	8.6%	
	2.00	39	30.5%	41	32.0%	
	3.00	9	7.0%	13	10.2%	
Does the nurse complete the safe medication administration checklist for preparing the medication?	1.00	15	11.7%	12	9.4%	
	2.00	30	23.4%	32	25.0%	
	3.00	18	14.1%	21	16.4%	
Does the nurse label the medication name, patient's name, and room number on the syringe and pump?	1.00	13	10.2%	12	9.4%	
	2.00	26	20.3%	29	22.7%	
	3.00	24	18.8%	24	18.8%	
Does the nurse prepare the medication by two people?	1.00	11	8.6%	11	8.6%	
	2.00	37	28.9%	32	25.0%	
	3.00	15	11.7%	18	14.1%	
	4.00	0	0.0%	4	3.1%	
Does the nurse double-check the unusual amount of medication?	1.00	18	14.1%	17	13.3%	
	2.00	34	26.6%	33	25.8%	
	3.00	11	8.6%	15	11.7%	
Does the nurse double-check the insulin dose?	1.00	18	14.1%	16	12.5%	
	2.00	27	21.1%	34	26.6%	
	3.00	18	14.1%	13	10.2%	
	4.00	0	0.0%	2	1.6%	
Does the nurse check the patient's allergy history before administering the medication?	1.00	18	14.1%	17	13.3%	
	2.00	30	23.4%	39	30.5%	
	3.00	15	11.7%	9	7.0%	
Is another nurse present during the preparation and administration of the medication?	1.00	34	26.6%	27	21.1%	
	2.00	28	21.9%	29	22.7%	
	3.00	1	0.8%	8	6.3%	
	4.00	0	0.0%	1	0.8%	

Medication performance after intervention
	Count	N%	
Does the nurse perform a complete patient identification and check the patient's wristband before administering the medication? (Patient Identification)?	1.00	6	4.7%	
	2.00	54	42.2%	
	3.00	68	53.1%	
Does the nurse prepare medications for more than one patient at a time?	1.00	7	5.5%	
	2.00	75	58.6%	
	3.00	35	27.3%	
	4.00	11	8.6%	
Does the nurse label the patient's name and room number on the medication container?	1.00	16	12.5%	
	2.00	71	55.5%	
	3.00	41	32.0%	
Does the nurse complete the safe medication administration checklist for preparing the medication?	1.00	10	7.8%	
	2.00	46	35.9%	
	3.00	71	55.5%	
	4.00	1	0.8%	
Does the nurse label the medication name, patient's name, and room number on the syringe and pump?	1.00	14	10.9%	
	2.00	44	34.4%	
	3.00	66	51.6%	
	4.00	4	3.1%	
Does the nurse prepare the medication by two people?	1.00	14	10.9%	
	2.00	47	36.7%	
	3.00	40	31.3%	
	4.00	27	21.1%	
Does the nurse double-check the unusual amount of medication?	1.00	19	14.8%	
	2.00	50	39.1%	
	3.00	56	43.8%	
	4.00	3	2.3%	
Does the nurse double-check the insulin dose?	1.00	27	21.1%	
	2.00	65	50.8%	
	3.00	19	14.8%	
	4.00	17	13.3%	
Does the nurse check the patient's allergy history before administering the medication?	1.00	33	25.8%	
	2.00	64	50.0%	
	3.00	24	18.8%	
	4.00	7	5.5%	
Is another nurse present during the preparation and administration of the medication?	1.00	24	18.8%	
	2.00	53	41.4%	
	3.00	41	32.0%	
	4.00	10	7.8%	


Medication performance after intervention
	Group	
	Intervention A	Control: B	
	Count	N %	Count	N %	
Does the nurse perform a complete patient identification and check the patient's wristband before administering the medication? (Patient Identification)?	1.00	0	0.0%	6	4.7%	
	2.00	31	24.2%	23	18.0%	
	3.00	32	25.0%	36	28.1%	
Does the nurse prepare medications for more than one patient at a time?	1.00	0	0.0%	7	5.5%	
	2.00	40	31.3%	35	27.3%	
	3.00	15	11.7%	20	15.6%	
	4.00	8	6.3%	3	2.3%	
Does the nurse label the patient's name and room number on the medication container?	1.00	6	4.7%	10	7.8%	
	2.00	31	24.2%	40	31.3%	
	3.00	26	20.3%	15	11.7%	
Does the nurse complete the safe medication administration checklist for preparing the medication?	1.00	0	0.0%	10	7.8%	
	2.00	15	11.7%	31	24.2%	
	3.00	47	36.7%	24	18.8%	
	4.00	1	0.8%	0	0.0%	
Does the nurse label the medication name, patient's name, and room number on the syringe and pump?	1.00	4	3.1%	10	7.8%	
	2.00	18	14.1%	26	20.3%	
	3.00	38	29.7%	28	21.9%	
	4.00	3	2.3%	1	0.8%	
Does the nurse prepare the medication by two people?	1.00	0	0.0%	14	10.9%	
	2.00	19	14.8%	28	21.9%	
	3.00	20	15.6%	20	15.6%	
	4.00	24	18.8%	3	2.3%	
Does the nurse double-check the unusual amount of medication?	1.00	7	5.5%	12	9.4%	
	2.00	25	19.5%	25	19.5%	
	3.00	28	21.9%	28	21.9%	
	4.00	3	2.3%	0	0.0%	
Does the nurse double-check the insulin dose?	1.00	9	7.0%	18	14.1%	
	2.00	28	21.9%	37	28.9%	
	3.00	11	8.6%	8	6.3%	
	4.00	15	11.7%	2	1.6%	
Does the nurse check the patient's allergy history before administering the medication?	1.00	14	10.9%	19	14.8%	
	2.00	30	23.4%	34	26.6%	
	3.00	13	10.2%	11	8.6%	
	4.00	6	4.7%	1	0.8%	
Is another nurse present during the preparation and administration of the medication?	1.00	11	8.6%	13	10.2%	
	2.00	26	20.3%	27	21.1%	
	3.00	20	15.6%	21	16.4%	
	4.00	6	4.7%	4	3.1%	


Satisfaction after intervention	Count	N %	
Were you satisfied with the teaching method?	1.00	17	13.3%	
	2.00	45	35.2%	
	3.00	66	51.6%	
Did this teaching method encourage you to participate in class?	1.00	15	11.7%	
	2.00	75	58.6%	
	3.00	38	29.7%	
Would you recommend this method for other educational materials?	1.00	23	18.0%	
	2.00	70	54.7%	
	3.00	30	23.4%	
	4.00	5	3.9%	
Did the teaching method make you more willing to participate in training sessions?	miss	1	0.8%	
	1.00	22	17.2%	
	2.00	55	43.0%	
	3.00	42	32.8%	
	4.00	8	6.3%	
How satisfied was your overall satisfaction with the teaching?	1.00	6	4.7%	
	2.00	54	42.2%	
	3.00	55	43.0%	
	4.00	13	10.2%	
Did you feel confused during the game stages and feel that the game was not easy?	miss	1	0.8%	
	1.00	16	12.5%	
	2.00	64	50.0%	
	3.00	37	28.9%	
	4.00	10	7.8%	
Did participating in the game create a sense of competition in you?	1.00	24	18.8%	
	2.00	43	33.6%	
	3.00	39	30.5%	
	4.00	22	17.2%	
Did you feel motivated to participate in the game again?	miss	2	1.6%	
	1.00	19	14.8%	
	2.00	59	46.1%	
	3.00	41	32.0%	
	4.00	7	5.5%	
Did you feel that the game transferred practical knowledge?	1.00	21	16.4%	
	2.00	59	46.1%	
	3.00	37	28.9%	
	4.00	11	8.6%	
Did the game succeed in establishing a connection between what you learned?	miss	2	1.6%	
	1.00	39	30.5%	
	2.00	60	46.9%	
	3.00	17	13.3%	
	4.00	10	7.8%	


Satisfaction after intervention	Group	
	Intervention A	Control: B	
	Count	N %	Count	N %	
Were you satisfied with the teaching method?	1.00	5	3.9%	12	9.4%	
	2.00	24	18.8%	21	16.4%	
	3.00	34	26.6%	32	25.0%	
Did this teaching method encourage you to participate in class?	1.00	4	3.1%	11	8.6%	
	2.00	37	28.9%	38	29.7%	
	3.00	22	17.2%	16	12.5%	
Would you recommend this method for other educational materials?	1.00	5	3.9%	18	14.1%	
	2.00	36	28.1%	34	26.6%	
	3.00	18	14.1%	12	9.4%	
	4.00	4	3.1%	1	0.8%	
Did the teaching method make you more willing to participate in training sessions?	miss	0	0.0%	1	0.8%	
	1.00	6	4.7%	16	12.5%	
	2.00	26	20.3%	29	22.7%	
	3.00	24	18.8%	18	14.1%	
	4.00	7	5.5%	1	0.8%	
How satisfied was your overall satisfaction with the teaching?	1.00	1	0.8%	5	3.9%	
	2.00	20	15.6%	34	26.6%	
	3.00	32	25.0%	23	18.0%	
	4.00	10	7.8%	3	2.3%	
 Did you feel confused during the game stages and feel that the game was not easy?	miss	0	0.0%	1	0.8%	
	1.00	4	3.1%	12	9.4%	
	2.00	30	23.4%	34	26.6%	
	3.00	20	15.6%	17	13.3%	
	4.00	9	7.0%	1	0.8%	
Did participating in the game create a sense of competition in you?	1.00	7	5.5%	17	13.3%	
	2.00	22	17.2%	21	16.4%	
	3.00	21	16.4%	18	14.1%	
	4.00	13	10.2%	9	7.0%	
 Did you feel motivated to participate in the game again?	miss	0	0.0%	2	1.6%	
	1.00	5	3.9%	14	10.9%	
	2.00	29	22.7%	30	23.4%	
	3.00	24	18.8%	17	13.3%	
	4.00	5	3.9%	2	1.6%	
 Did you feel that the game transferred practical knowledge?	1.00	3	2.3%	18	14.1%	
	2.00	32	25.0%	27	21.1%	
	3.00	20	15.6%	17	13.3%	
	4.00	8	6.3%	3	2.3%	
 Did the game succeed in establishing a connection between what you learned?	miss	1	0.8%	1	0.8%	
	1.00	14	10.9%	25	19.5%	
	2.00	29	22.7%	31	24.2%	
	3.00	10	7.8%	7	5.5%	
	4.00	9	7.0%	1	0.8%	
