# Supplementary material for: The effect of gamification on the medication knowledge, performance and satisfaction of nurses in continued medical education: A quasi-experimental study
Source: PLoS One. 2025 Sep 4;20(9):e0331372. doi: 10.1371/journal.pone.0331372 (PMC12410705; doi:10.1371/journal.pone.0331372)
Supplement: Peer review history

# Peer review: Decision Letter 0

**DOI:** 10.1371/journal.pone.0331372.r001

**Authors:** Amir Karimi

28 Jul 2025

Thank you for submitting your manuscript to PLOS ONE. After careful consideration, we feel that it has merit but does not fully meet PLOS ONE’s publication criteria as it currently stands. Therefore, we invite you to submit a revised version of the manuscript that addresses the points raised during the review process.

Please submit your revised manuscript by Sep 11 2025 11:59PM. If you will need more time than this to complete your revisions, please reply to this message or contact the journal office at plosone@plos.org . When you're ready to submit your revision, log on to https://www.editorialmanager.com/pone/ and select the 'Submissions Needing Revision' folder to locate your manuscript file.

- A rebuttal letter that responds to each point raised by the academic editor and reviewer(s). You should upload this letter as a separate file labeled 'Response to Reviewers'.
- A marked-up copy of your manuscript that highlights changes made to the original version. You should upload this as a separate file labeled 'Revised Manuscript with Track Changes'.
- An unmarked version of your revised paper without tracked changes. You should upload this as a separate file labeled 'Manuscript'.

If applicable, we recommend that you deposit your laboratory protocols in protocols.io to enhance the reproducibility of your results. Protocols.io assigns your protocol its own identifier (DOI) so that it can be cited independently in the future. For instructions see: https://journals.plos.org/plosone/s/submission-guidelines#loc-laboratory-protocols . Additionally, PLOS ONE offers an option for publishing peer-reviewed Lab Protocol articles, which describe protocols hosted on protocols.io. Read more information on sharing protocols at https://plos.org/protocols?utm_medium=editorial-email&utm_source=authorletters&utm_campaign=protocols .

We look forward to receiving your revised manuscript.

Kind regards,

Amir Karimi, PhD

Academic Editor

PLOS ONE

Journal Requirements:

When submitting your revision, we need you to address these additional requirements.

1. Please ensure that your manuscript meets PLOS ONE's style requirements, including those for file naming. The PLOS ONE style templates can be found at

https://journals.plos.org/plosone/s/file?id=wjVg/PLOSOne_formatting_sample_main_body.pdf and

https://journals.plos.org/plosone/s/file?id=ba62/PLOSOne_formatting_sample_title_authors_affiliations.pdf

2. We note that your Data Availability Statement is currently as follows: [All relevant data are within the manuscript and its Supporting Information files.]

Please confirm at this time whether or not your submission contains all raw data required to replicate the results of your study. Authors must share the “minimal data set” for their submission. PLOS defines the minimal data set to consist of the data required to replicate all study findings reported in the article, as well as related metadata and methods (https://journals.plos.org/plosone/s/data-availability#loc-minimal-data-set-definition).

For example, authors should submit the following data:

- The values behind the means, standard deviations and other measures reported;

- The values used to build graphs;

- The points extracted from images for analysis.

Authors do not need to submit their entire data set if only a portion of the data was used in the reported study.

If your submission does not contain these data, please either upload them as Supporting Information files or deposit them to a stable, public repository and provide us with the relevant URLs, DOIs, or accession numbers. For a list of recommended repositories, please see https://journals.plos.org/plosone/s/recommended-repositories.

If there are ethical or legal restrictions on sharing a de-identified data set, please explain them in detail (e.g., data contain potentially sensitive information, data are owned by a third-party organization, etc.) and who has imposed them (e.g., an ethics committee). Please also provide contact information for a data access committee, ethics committee, or other institutional body to which data requests may be sent. If data are owned by a third party, please indicate how others may request data access.

3. Your ethics statement should only appear in the Methods section of your manuscript. If your ethics statement is written in any section besides the Methods, please move it to the Methods section and delete it from any other section. Please ensure that your ethics statement is included in your manuscript, as the ethics statement entered into the online submission form will not be published alongside your manuscript.

4. We are unable to open your Supporting Information file [gamefication 2.sav]. Please kindly revise as necessary and re-upload.

5. Please include captions for your Supporting Information files at the end of your manuscript, and update any in-text citations to match accordingly. Please see our Supporting Information guidelines for more information: http://journals.plos.org/plosone/s/supporting-information .

6. If the reviewer comments include a recommendation to cite specific previously published works, please review and evaluate these publications to determine whether they are relevant and should be cited. There is no requirement to cite these works unless the editor has indicated otherwise. 

7. Please review your reference list to ensure that it is complete and correct. If you have cited papers that have been retracted, please include the rationale for doing so in the manuscript text, or remove these references and replace them with relevant current references. Any changes to the reference list should be mentioned in the rebuttal letter that accompanies your revised manuscript. If you need to cite a retracted article, indicate the article’s retracted status in the References list and also include a citation and full reference for the retraction notice.

Additional Editor Comments:

Hello, dear authors

Please check the journal website for the style of writing articles, numbering, etc.

Recheck the numbers of tables and figures.

Try to write concisely, clearly, and documented.

I hope you will attach the file of similarity finding and the referees' responses completely and scientifically.

Thank you very much

[Note: HTML markup is below. Please do not edit.]

Reviewers' comments:

Reviewer's Responses to Questions

**Comments to the Author**

1. Is the manuscript technically sound, and do the data support the conclusions?

Reviewer #1: Yes

Reviewer #2: Partly

Reviewer #3: Yes

**********

2. Has the statistical analysis been performed appropriately and rigorously?

Reviewer #1: Yes

Reviewer #2: Yes

Reviewer #3: Yes

**********

3. Have the authors made all data underlying the findings in their manuscript fully available?

The PLOS Data policy

Reviewer #1: Yes

Reviewer #2: Yes

Reviewer #3: Yes

**********

4. Is the manuscript presented in an intelligible fashion and written in standard English?

Reviewer #1: No

Reviewer #2: Yes

Reviewer #3: Yes

**********

Reviewer #1: Dear Authors,

Thank you for your submission.

The authors investigated the effect of gamification on the medication knowledge, performance and satisfaction of nurses in continued medical education. The study used a quasi-experimental design. Nevertheless, some aspects can be improved to enhance the paper's readability. Proofreading is recommended as well.

Additional comments:

1- Method

Participants:

• The following exclusion criterion is not required as it does happen by default: “The exclusion criteria included not participating in the education session”

• Although the sample was convenient, the sample size is recommended to be calculated based on a rigorous statistical method.

• Data analysis:

The preliminary p-value should be added.

The inferential statistics used should be described.

Figures 1 and 2: The quality and resolution are poor.

2. Results:

• Table 3, please provide details about the mean of satisfaction before and after the intervention.

3. Discussion:

• Presenting the findings of the previous studies was narrative and not well synthesized in the results. The researchers are advised to consider this when revising the discussion section.

• The researchers did not reflect on their findings in comparison to previous studies, using their own words. This necessitates further improvement of the discussion section.

• The researchers are recommended to present recommendations for future research.

• The limitations of the study should be more specific in describing whether the internal validity of the survey was threatened or not.

• The strengths of the study should be presented.

Sincerely,

Reviewer #2: While the abstract mentions Kahoot, it might be beneficial to briefly state what Kahoot is (e.g., "a competitive educational software") in the abstract for readers unfamiliar with it, before explaining its game-based nature in the conclusion.

Consider adding a brief section on the practical implications of the findings for healthcare educators and administrators.

Ensure consistent use of "P-value" or "p-value" throughout the manuscript.

Double-check for any minor grammatical errors or typos.

Reviewer #3: Dear authors,

Congratulations on your work. Indeed, any innovative strategy in teaching and learning processes must be thoroughly studied for its implementation. Your study can contribute to this evidence.

Analyzing your submission in more detail...

Starting with the abstract, it seems good. Clearly indicative of the study. I suggest, however, changes to the keywords for health descriptors. For example, Mesh terms. Consider changing: "game base education"; "Performance" by "work performance" ; "Satisfaction" by "job satisfaction" or "personal satisfaction".

The introduction is consistent with the work. It introduces the topic clearly and accurately.The references used are current and relevant to frame the theme. Revise the word prescribe in the second line of introduction "nurses must prescribe the correct medicine". We think the verb administer would be better.

As far as the methods are concerned, in general they look good. However, they should be improved:

-Do not mention the name of the institution where the study was developed, as it identifies the services;

-Put "Convinience method" in lower case.

-Clarify the entire population of nurses in these services. Were there no nurses on holiday or absent due to illness? Did the nurses with management functions take part in the study?

-Explain how the randomisation method was carried out. Did you use any software? Did you draw lots?

-Clarify whether the Kahoot was carried out at the end of each session or only at the end of each session. Does this mean that there are more sessions, or were they carried out in the five planned sessions? Please clarify...

Did the participants know whether or not they were in the intervention group?

- Clarify whether the instrument in part 2 of the questionnaire was developed by Salami et al. (2019) or by Wakefield et al. (2005) Wakefield BJ, Uden-Holman T, Wakefield DS. Development and validation of the medication administration error reporting survey. In: Advances in Patient Safety: From Research to Implementation (Volume 4: Programs, Tools, and Products). Rockville (MD): Agency for Healthcare Research and Quality (US); 2005.

-Explain in this sentence "In order to check face validity and qualitative

content validity, the questionnaires were given to 10 faculty members and clinical

nurses, and after collecting their opinions, necessary modifications were made to

the questionnaires." whether they used the Delphi methodology; what criteria were used for selecting the panel of experts, if so considered; what changes were made to the initial questionnaire.

With regard to the results, consider putting the contents of the tables on a single page.

Do not use the author's name with et al in sentences.

The discussion is well done. Improve the writing according to the suggestions given.

In conclusion, consider the need to develop further studies that deepen the motivations of trainees and trainers in the use of new pedagogical strategies such as gamification, among others.

**********

what does this mean? ). If published, this will include your full peer review and any attached files.

If you choose “no”, your identity will remain anonymous but your review may still be made public.

**Do you want your identity to be public for this peer review?** For information about this choice, including consent withdrawal, please see our Privacy Policy

Reviewer #1: **Yes: ** Saleh Al Omar

Reviewer #2: No

Reviewer #3: **Yes: ** Maria Cristina Bompastor Augusto

**********

[NOTE: If reviewer comments were submitted as an attachment file, they will be attached to this email and accessible via the submission site. Please log into your account, locate the manuscript record, and check for the action link "View Attachments". If this link does not appear, there are no attachment files.]

While revising your submission, please upload your figure files to the Preflight Analysis and Conversion Engine (PACE) digital diagnostic tool, https://pacev2.apexcovantage.com/ . PACE helps ensure that figures meet PLOS requirements. To use PACE, you must first register as a user. Registration is free. Then, login and navigate to the UPLOAD tab, where you will find detailed instructions on how to use the tool. If you encounter any issues or have any questions when using PACE, please email PLOS at figures@plos.org

# Author comment: Author response to Decision Letter 1

**DOI:** 10.1371/journal.pone.0331372.r002

6 Aug 2025

Dear Editor

We appreciate your valuable feedback, as well as that of the reviewers. The necessary revisions have been completed.

Kind regards

# Editor report: Decision Letter 1

**DOI:** 10.1371/journal.pone.0331372.r003

**Authors:** Amir Karimi

15 Aug 2025

The Effect of Gamification on the Medication Knowledge, Performance and Satisfaction of Nurses in Continued Medical Education: a Quasi-Experimental Study

PONE-D-25-29769R1

Dear Dr. Ghafouri,

We’re pleased to inform you that your manuscript has been judged scientifically suitable for publication and will be formally accepted for publication once it meets all outstanding technical requirements.

Within one week, you’ll receive an e-mail detailing the required amendments. When these have been addressed, you’ll receive a formal acceptance letter and your manuscript will be scheduled for publication.

An invoice will be generated when your article is formally accepted. Please note, if your institution has a publishing partnership with PLOS and your article meets the relevant criteria, all or part of your publication costs will be covered. Please make sure your user information is up-to-date by logging into Editorial Manager at Editorial Manager®  and clicking the ‘Update My Information' link at the top of the page. For questions related to billing, please contact billing support .

If your institution or institutions have a press office, please notify them about your upcoming paper to help maximize its impact. If they’ll be preparing press materials, please inform our press team as soon as possible -- no later than 48 hours after receiving the formal acceptance. Your manuscript will remain under strict press embargo until 2 pm Eastern Time on the date of publication. For more information, please contact onepress@plos.org.

Kind regards,

Amir Karimi, PhD

Academic Editor

PLOS ONE

Additional Editor Comments (optional):

Reviewers' comments:

# Editor report: Acceptance letter

**DOI:** 10.1371/journal.pone.0331372.r004

**Authors:** Amir Karimi

PONE-D-25-29769R1

PLOS ONE

Dear Dr. Ghafouri,

I'm pleased to inform you that your manuscript has been deemed suitable for publication in PLOS ONE. Congratulations! Your manuscript is now being handed over to our production team.

At this stage, our production department will prepare your paper for publication. This includes ensuring the following:

* All references, tables, and figures are properly cited

* All relevant supporting information is included in the manuscript submission,

* There are no issues that prevent the paper from being properly typeset

You will receive further instructions from the production team, including instructions on how to review your proof when it is ready. Please keep in mind that we are working through a large volume of accepted articles, so please give us a few days to review your paper and let you know the next and final steps.

Lastly, if your institution or institutions have a press office, please let them know about your upcoming paper now to help maximize its impact. If they'll be preparing press materials, please inform our press team within the next 48 hours. Your manuscript will remain under strict press embargo until 2 pm Eastern Time on the date of publication. For more information, please contact onepress@plos.org.

You will receive an invoice from PLOS for your publication fee after your manuscript has reached the completed accept phase. If you receive an email requesting payment before acceptance or for any other service, this may be a phishing scheme. Learn how to identify phishing emails and protect your accounts at https://explore.plos.org/phishing.

If we can help with anything else, please email us at customercare@plos.org.

Thank you for submitting your work to PLOS ONE and supporting open access.

Kind regards,

PLOS ONE Editorial Office Staff

on behalf of

Dr. Amir Karimi

Academic Editor

PLOS ONE
